# Marek's Disease Virus and Reticuloendotheliosis Virus Coinfection Enhances Viral Replication and Alters Cellular Protein Profiles

**DOI:** 10.3389/fvets.2022.854007

**Published:** 2022-03-22

**Authors:** Xusheng Du, Defang Zhou, Jing Zhou, Jingwen Xue, Ziqiang Cheng

**Affiliations:** College of Veterinary Medicine, Shandong Agricultural University, Tai'an, China

**Keywords:** proteomic analysis, coinfection, Marek's disease virus, reticuloendotheliosis virus, TMT

## Abstract

Coinfection with Marek's disease virus (MDV) and reticuloendotheliosis virus (REV) causes synergistic pathogenic effects and serious losses to the poultry industry. However, whether there is a synergism between the two viruses in viral replication and the roles of host factors in regulating MDV and REV coinfection remains elusive. In this study, we found that MDV and REV coinfection increased viral replication in coinfected cells as compared to a single infection in a limited period. Further, we explore the host cell responses to MDV and REV coinfection using tandem mass tag (TMT) peptide labeling coupled with liquid chromatography–tandem mass spectrometry (LC-MS/MS). Compared with MDV/REV-infected cells, 38 proteins increased (fold change > 1.2) and 60 decreased (fold change < 0.83) their abundance in MDV and REV coinfected cells. Differentially accumulated proteins (DAPs) were involved in important biological processes involved in the immune system process, cell adhesion and migration, cellular processes, and multicellular organismal systems. STRING analysis found that IRF7, MX1, TIMP3, and AKT1 may be associated with MDV and REV synergistic replication in chicken embryo fibroblasts (CEFs). Western blotting analysis showed that the selected DAPs were identical to the quantitative proteomics data. Taken together, we verified that MDV and REV can synergistically replicate in coinfected cells and revealed the host molecules involved in it. However, the synergistic pathogenesis of MDV and REV needs to be further studied.

## Introduction

Marek's disease virus (MDV), an oncogenic alpha-herpesvirus, causes Marek's disease (MD) (1). Reticuloendotheliosis virus (REV), an oncogenic and immunosuppressive retrovirus, causes reticuloendotheliosis (RE) (2). Two contagious, immunosuppressive, and oncogenic diseases in chickens affect poultry production, and mixed infection of MDV and REV has become an important epidemiologic situation worldwide (3–5). Synergistic pathogenicity usually occurred between MDV and REV, as MDV and REV coinfection significantly increased disease severity (6, 7). The common occurrence of REV among MDV-infected chickens may be linked to contaminated vaccine stocks (8), and MDV due to REV long terminal repeat (LTR) recombinant increased horizontal transmission (9, 10). Generally, virus pathogenicity is related to the ability of viral replication. MDV and REV genome load contributes to our understanding of the pathogenesis of two virus infections. However, the effects of coinfection with MDV and REV on replication remain unknown.

To further understand the synergistic pathogenesis of MDV and REV coinfection, research on virus–host interaction is critical. Virus infection can affect host cell morphology, the cytoskeleton, the cell cycle, transcription and translation patterns, and innate immune responses of the host, the apoptosis pathway (11, 12). The morphological and functional changes are associated with significant changes in the patterns of expression of host cells (13, 14). Consequently, information on proteome changes in the host following MDV and REV coinfection may be crucial to understanding the host response to viral pathogenesis. In recent years, the development of comparative proteomics has facilitated the study of host cellular responses to pathogen infection (15). Proteomics technologies have been used to characterize the pathogenesis of MDV and REV infection, recently. Protein expression of chicken embryo fibroblast (CEF) cells infected with MDV has been studied using a modified MudPIT analysis involving strong cation exchange chromatography and microcapillary reversed-phase liquid chromatography–tandem mass spectrometry (LC-MS/MS) (16). Isobaric tags for relative and absolute quantification (iTRAQ) approach were used to analyze the protein profile of REV-infected CEFs (17). Among the current proteomics methods, tandem mass tag (TMT) quantitative proteomics techniques, the highly sensitive proteomic platform based on the isobaric labels TMTs as one of the most robust proteomics techniques, are useful for the analysis of infection-associated proteins (18).

However, to the best of our knowledge, no previous study has analyzed the proteomic changes in MDV/REV coinfected CEF cells. We examined the effects of coinfection on REV and MDV replication and used the TMT-labeling quantitative detection technique to quantify CEF cells proteins that are differentially expressed after infection by REV alone, MDV alone, or coinfection by both agents. A total of 98 common differentially expressed proteins were identified at 48 h post-infection (hpi). Analysis of these altered expression proteins might provide fundamental information for the study of virus–host interactions and the molecular basis underlying MDV and REV synergistic replication *in vitro*.

## Materials and Methods

### Cells and Viruses

The Md5 strain of MDV (10^3^ PFU/0.2 ml) and the single-nucleotide variant (SNV) strain of REV (10^4^ TCID_50_/0.2 ml) were maintained in our laboratory (19, 20). CEF cells were maintained in Dulbecco's Modified Eagle's Medium (DMEM; Sigma, CA, USA) supplemented with 10% fetal bovine serum (FBS; HyClone, UT, USA) in a 5% CO_2_ incubator at 37°C. The replication of MDV and REV was measured using the pfu and TCID_50_ methods in the CEF cells at various time points, respectively.

### Overview of the Experimental Design

**Figure 1:**
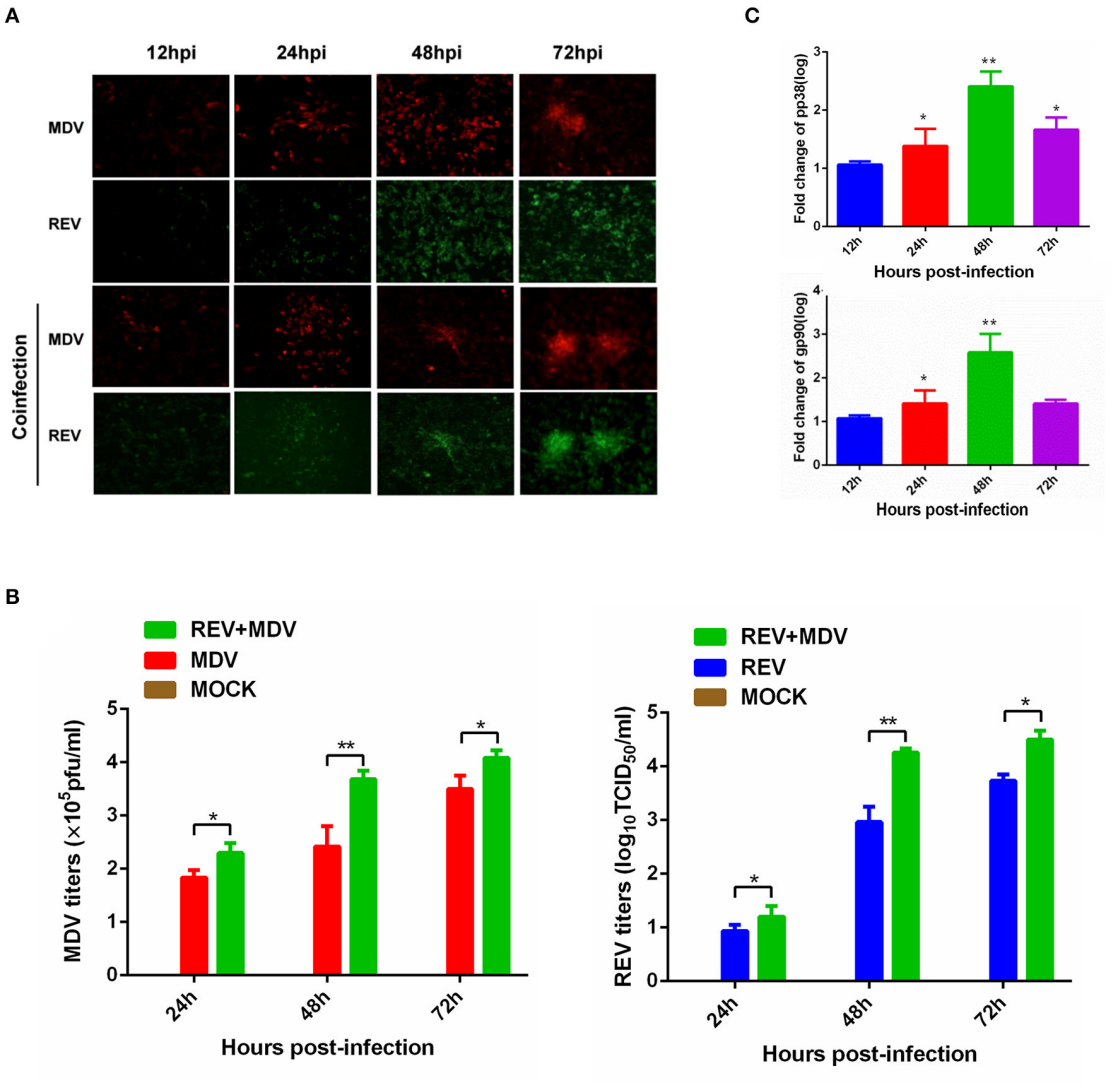
Synergistic infection of MDV and REV increases the two viruses' replication in CEF. **(A)** Detection of MDV-pp38 and REV-gp90 in infected CEFs was visualized by IFA from 24 to 72 hpi. FITC-labeled goat anti-mouse IgG (for REV) (green) and Cy3-labeled goat anti-rabbit IgG (for MDV) (red) were used as the secondary antibodies in the assay. **(B)** The MDV and REV viral titers were tested from 24 to 72 hpi. **(C)** Differential expression fold change of MDV pp38 and REV gp90 genes in the MDV and REV coinfected cells compared to the single-infected control group. The fold change between both groups is shown using a logarithmic scale. Data are presented as the mean ± SD from three independent experiments. *p < 0.05; **p < 0.01. MDV, Marek's disease virus; REV, reticuloendotheliosis virus; CEF, chicken embryo fibroblast; IFA, immunofluorescence assay; FITC, fluorescein isothiocyanate.

To study the effects of coinfection *in vitro*, CEF cell monolayers were subjected to one of four conditions (Figure 1D): (a) non-infected cells served as negative controls; (b) cells infected with REV only; (c) cells infected with MDV only; and d) cells infected with REV for 24 h and then infected with MDV (coinfected group). The multiplicity of infection (MOI) and incubation time of viruses were selected to allow replication while causing minimal damage to CEF cells. Confocal imaging, Western blotting, and qRT-PCR were used to detect viral infection and proliferation. Every sample was repeated in three technical replicates, and each experiment was conducted three times. The above samples were prepared for comparative proteomic analysis at the appropriate time intervals.

### mRNA Quantitation by qRT-PCR

Total cellular RNA was extracted from the MDV-infected, REV-infected, MDV/REV coinfected, and mock-infected CEF cells using RNAiso Plus (TaKaRa, Dalian, China) following the manufacturer's instructions. cDNA was prepared from the RNA using ReverTra Ace qPCR RT Master Mix (TOYOBO, Shanghai, China) and used as a template for quantitative reverse transcription PCR (qRT-qPCR). Whereafter qRT-PCR was performed using the SYBR Premix Ex Taq™ II Kit (TaKaRa) on a Roche LightCycler 96 system (Roche, Basel, Switzerland). Real-time quantitative PCR (qPCR) detection was performed as previously described (20). Specific primers for amplifying various genes were as follows: for GAPDH mRNA analysis, 5′-GAACATCATCCCAGCGTCCA-3′ (forward) and 5′-GGTCATAAGTCCCTCCACGA-3′ (reverse) were used; for REV gp90 analysis, 5′-GGCATCAATCGTACCCGACA-3′ (forward) and 5′-GGGGGATAAACTGGACTGCC-3′ (reverse) were used; for MDV pp38 analysis, 5′-GCTGCAGCTGTCCATTTTCC-3′ (forward) and 5′-TACAGTGTAGCCGTACCCGA-3′ (reverse) were used. GAPDH was employed as an internal reference gene. The relative expression level of each mRNA was calculated by the 2^−Δ*Δct*^ method. Three independent biological replicates were performed for each gene.

### Immunofluorescence Assay and Confocal Imaging

CEF cells cultured in 24-well culture plates and 15-mm culture dishes were infected and coinfected with MDV and REV. For immunofluorescence assay (IFA), cells were first fixed with 4% paraformaldehyde for 30 min and permeabilized with 0.1% Triton X-100 in phosphate-buffered saline (PBS) for 15 min, followed by blocking with 5% bovine serum albumin in PBS for 1 h. Whereafter, the cells were incubated with mouse anti-gp90 and fluorescein isothiocyanate (FITC)-labeled goat anti-mouse IgG (for REV) or rabbit anti-pp38 and Cy3-labeled goat anti-rabbit IgG (for MDV) diluted in PBS for 1 h. For confocal imaging, the above cells were examined using an SP8 confocal laser scanning microscope (CLSM) (Leica, Wetzlar, Germany). The overlap of the two colors of fluorescent markers appears yellow. The nuclei of all the infected cells were stained by DAPI.

### Western Blotting

Total protein lysates were isolated from treated CEF cells using a lysis buffer [pH 7.6, 0.1 mmol/L of NaCl, 0.01 mmol/L of Tris-HCl, 0.001 mol/L of ethylene diamine tetraacetic acid (EDTA), pH 8.0, 1 μg/ml of aprotinin, 100 μg/ml of phenylmethylsulfonyl fluoride (PMSF)]. The protein concentrations were measured by BCA Protein Assay Kit (PIERCE, Rockford, IL, USA). The proteins were separated by 10% sodium dodecyl sulfate–polyacrylamide gel electrophoresis (SDS-PAGE) and transferred to polyvinylidene fluoride membranes (Millipore, Billerica, USA), which were blocked for 2 h in 5% defatted milk in Tris-buffered saline containing Tween-20. The membranes were incubated at 37°C for 60 min with rabbit polyclonal antibody to TIMP3 (Abcam, Cambridge, UK), rabbit polyclonal antibody to AKT1 (BIOSS, Beijing, China), rabbit polyclonal antibody to MX1 (ProteinTech Group, Chicago, IL, USA), rabbit polyclonal antibody to IRF7 (BIOSS, Beijing, China), mouse monoclonal antibody anti-REV gp90, and rabbit polyclonal anti-MDV pp38. After being washed three times with 0.05% TBST, the membranes were incubated at 37°C for 60 min with horseradish peroxidase (HRP)-conjugated goat anti-rabbit IgG or goat anti-mouse IgG (BIOSS, Beijing, China). The results were detected using the ECL Detection Kit (Vazyme, Nanjing, China). β-Actin was used as an internal control.

### Protein Sample Preparation, Trypsin Digestion, and Tandem Mass Tag Labeling

The MDV-infected, REV-infected, MDV/REV coinfected, and mock-infected CEF cells were washed with PBS and collected in lysis buffer (8 M of urea, 1% Protease Inhibitor Cocktail). Three biological replicates of the sample were sonicated on ice using a high-intensity ultrasonic processor (Scientz, Ningbo, China) and then centrifuged at 4°C for 10 min at 12,000 *g*. The supernatant was collected, and total protein concentration was determined using a BCA kit (Beyotime, Shanghai, China).

For digestion, the dithiothreitol (5 mM) was added to reduce protein solution at 56°C for 30 min, and iodoacetamide was used to alkylate for 15 min at room temperature. After that, trypsin was added overnight for the first digestion, 1:50 trypsin-to-protein mass ratio; then for the second digestion, trypsin was added at 4 h, 1:100 trypsin-to-protein mass ratio.

After trypsin digestion, the peptide was desalted and then processed with a TMT kit (Thermo Fisher Scientific, Waltham, MA, USA) according to the manufacturer's protocol.

### High-Performance Liquid Chromatography Fractionation and Liquid Chromatography–Tandem Mass Spectrometry Analysis

The tryptic peptides were fractionated by Agilent 300 Extend C18 column (Agilent, Santa Clara, CA, USA) using a high-performance liquid chromatography (HPLC) system (Thermo Fisher Scientific). For LC-MS/MS analysis, the tryptic peptides were dissolved and analyzed on an EASY-nLC 1000 UPLC system (Thermo Fisher Scientific, Waltham, MA, USA) at a constant flow rate of 450 nl/min. Solvent A (0.1% formic acid) was used to dissolve tryptic peptides, held at solvent B, and increased gradient from 8 to 23% with 0.1% formic acid in 90% acetonitrile.

MS/MS was performed on Q Exactive™ HF-X system (Thermo Fisher Scientific, Waltham, MA, USA). MS1 spectra were collected in the 2.0 kV electrospray voltage and in the range 350–1,600 *m*/*z*. For MS/MS, noise-contrastive estimation (NCE) setting as 28, the selected peptides were detected in the Orbitrap at a resolution of 17,500. A data-dependent procedure that alternated between one MS scan followed by 20 MS/MS scans with 15.0 s dynamic exclusion. Automatic gain control (AGC) was set at 5E4. Fixed first mass was set as 100 *m*/*z*. The HPLC fractionation and LC-MS/MS analysis in our research are supported by Jingjie PTM BioLabs (Hangzhou, China).

### Database Search

The MS/MS data were submitted to the Maxquant search engine (v.1.5.2.8) for data analysis. MS/MS spectra were searched against the Uniprot Gallus database (27535 sequences, downloaded on May 30, 2021) concatenated with reverse decoy database. Two missing cleavages were allowed in Trypsin/P with 20-ppm first search and 5-ppm main search, and the fragment ion mass tolerance was 0.02 Da. The false discovery rate (FDR) calculation was adjusted to <1%, and the minimum score was set to >40. For quantification, the unused value was >1.2, and the proteins had at least one unique peptide.

### Bioinformatics Analysis

The biological interpretation and function of identified proteins were analyzed using Gene Ontology (GO) annotation (www.http://www.ebi.ac.uk/GOA/) and Kyoto Encyclopedia of Genes and Genomes (KEGG) pathway mapping through KEGG mapper web server (http://www.genome.jp/kegg/tool/map_pathway2.html). GO enrichment and KEGG pathway enrichment were performed using a double-tailed Fisher's precision test. The GO and KEGG pathways with a corrected *p*-value < 0.05 were considered significant. The protein–protein interaction network was analyzed by the STRING database (http://string.embl.de/).

### Statistical Analysis

Data were analyzed by one-way repeated-measures ANOVA and least significance difference (LSD), which was considered statistically significant when *p* < 0.05.

## Results

### Coinfection of Marek's Disease Virus and Reticuloendotheliosis Virus Increases the Virus Replication in Chicken Embryo Fibroblasts

MDV and REV infections were confirmed using IFA, as REV does not induce cytopathic effects (CPEs) in CEFs. In MDV-infected (MOI = 1) cells, fluorescence intensity was observed obviously at 24 and 48 hpi. However, the cell death was present at 72 hpi (Supplementary Figure 1). In REV (MOI = 1) infected cells, weak fluorescence was observed beginning at 48 hpi (Supplementary Figure 1). To understand the effect of coinfection on MDV and REV replication, according to the above experimental results, the CEFs were first infected with REV (MOI = 1) or mock-infected for 24 h and then infected with the MDV (MOI = 0.1) for 12, 24, 48, and 72 h. The results of IFA showed that the fluorescence signal was more intense in MDV and REV coinfected cells compared to MDV/REV-infected cells. Meanwhile, in MDV and REV coinfected cells, the CPE was observed earlier and more severe than that of MDV-infected cells. The viral titers of MDV and REV were quantified by plaque assay and the TCID_50_ assay. As the results show, the replication rate of MDV or REV was higher at 24 hpi (*p* < 0.05), 48 hpi (*p* < 0.01), and 72 hpi (*p* < 0.05) in the MDV and REV coinfected group compared to the MDV/REV-infected control group (Figure 1B). Consistently, the results of qRT-PCR showed that MDV pp38 and REV gp90 mRNA expressed levels increased gradually and reached a peak at 48 h in MDV and REV coinfected cells (Figure 1C). All the results suggested that MDV and REV synergistically increase viral replication in CEF cells.

### Marek's Disease Virus and Reticuloendotheliosis Virus Are Localized in the Same Cell

**Figure 2:**
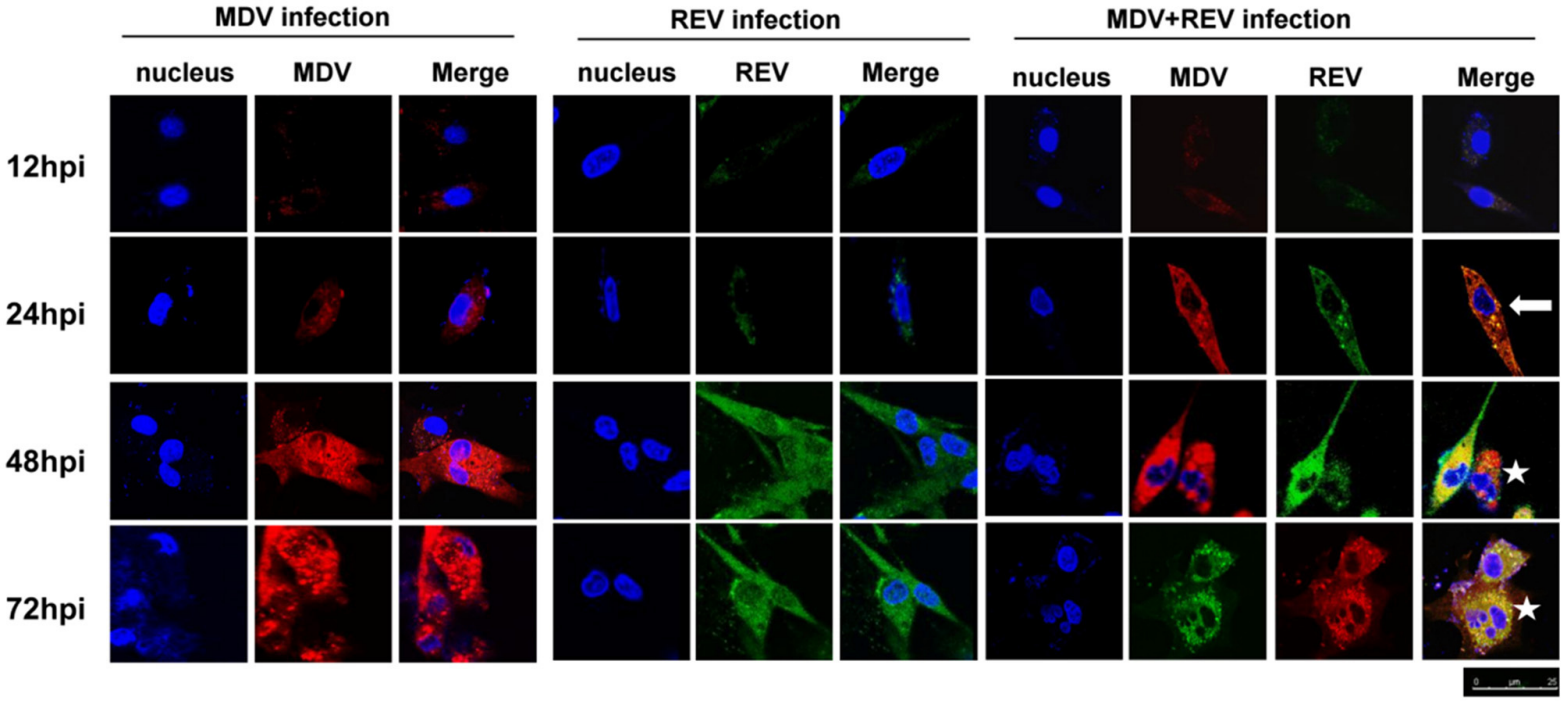
Protein expression and localization of MDV and REV examined by confocal laser scanning microscope (CLSM). The proteins of MDV and REV were co-localized in cytoplasm (arrowhead). The coinfected cells showed more cytopathy (CPE) (star) than single-infected cells. Samples stained with pp38 or gp90 antibodies as well as Cy3-labeled goat anti-rabbit IgG (for MDV) (red) or FITC-labeled goat anti-mouse IgG (for REV) (green) antibody in the assay. The areas of colocalization are shown in yellow. Cell nuclei (blue) were stained with DAPI. MDV, Marek's disease virus; REV, reticuloendotheliosis virus; CLSM, confocal laser scanning microscope; CPE, cytopathic effect; FITC, fluorescein isothiocyanate.

CLSM was performed to assess whether MDV and REV can replicate in the same CEF cell and whether MDV and REV coinfection could affect the subcellular localization of MDV or REV proteins using MDV pp38-specific and REV gp90-specific antibodies. The results of the CLSM assay showed that the MDV and REV signals could be detected simultaneously in the same cells, indicating that MDV and REV could coexist in or coinfect a cell. Dynamic fluorescent signal analysis showed that MDV pp38 protein and REV gp90 protein accumulated significantly higher and that the CPE appeared earlier in coinfected cells than that of single-infected cells (Figure 2). The data indicated that MDV and REV could replicate in the same cells and throw light on the possible synergistic mechanism of MDV and REV.

For further proteomic analysis, a higher proportion of infected cells and avoiding an excessive CPE are necessary. We selected 48 hpi as the time point to prepare samples under our infection conditions according to the results of Figure 1. In addition, a significant difference (*p* < 0.01) in titers of the MDV/REV progeny between MDV-REV coinfected cells and MDV/REV-infected cells may indicate significant changes in the patterns of expression of host cells at 48 hpi (Figure 1B).

### Global Proteome Analysis Upon Single and Coinfection of Marek's Disease Virus and Reticuloendotheliosis Virus

**Figure 3:**
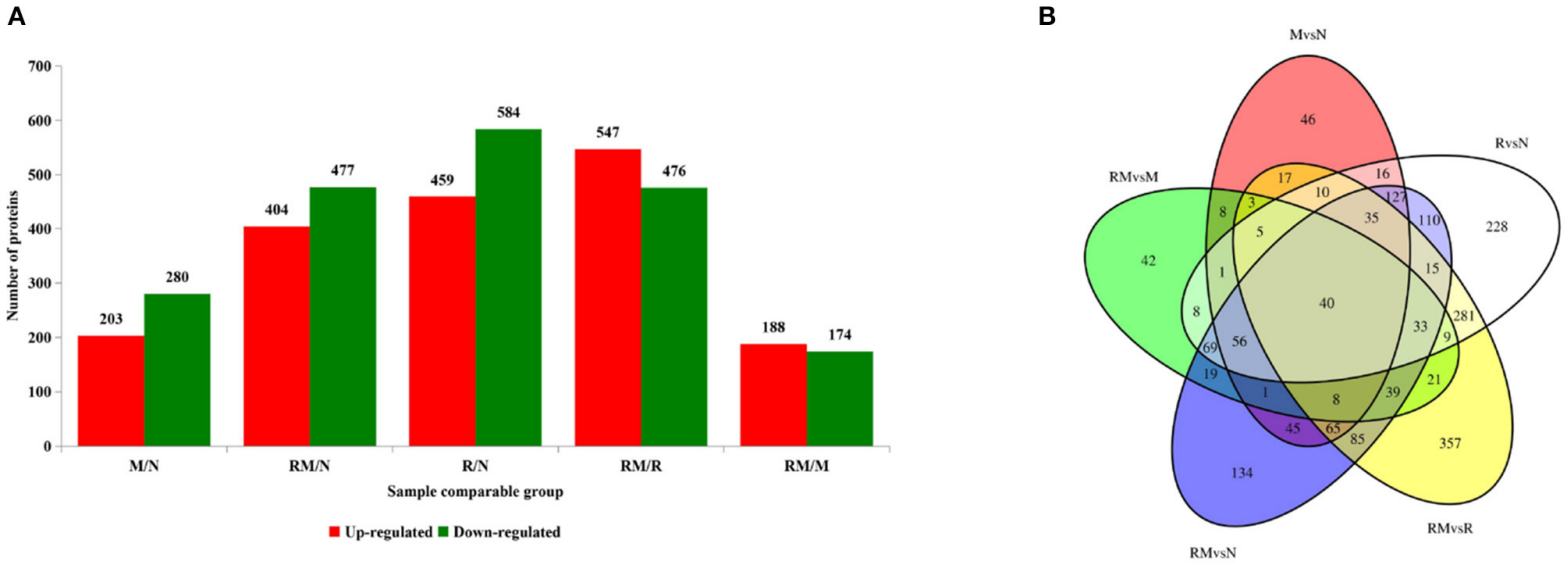
Distribution of differentially accumulated proteins (DAPs). **(A)** Number of upregulated (red) and downregulated (green) DAPs in M vs. N, R vs. N, RM vs. N, RM vs. M, and RM vs. R. **(B)** Venn diagram to show the distribution of DAPs between RM vs. N (blue circle), RM vs. R (yellow circle), RM vs. M (green circle), R vs. N (white circle), and M vs. N (red circle). N, mock-infected CEF cells; R, REV-infected CEF cells; M, MDV-infected CEF cells; RM, REV and MDV coinfected CEF cells. CEF, chicken embryo fibroblast; REV, reticuloendotheliosis virus; MDV, Marek's disease virus.

Extraction of total proteins from mock-infected CEF cells (N), REV-infected CEF cells (R), MDV-infected CEF cells (M), and REV and MDV coinfected CEF cells (RM) at 48 hpi. A total of 3,515 proteins were obtained from LC-MS/MS proteomic analysis, and 2407 proteins were identified (Supplementary Table 1). A total of 1,043, 483, and 881 detected proteins were identified in comparing R versus N (459 and 584 proteins were upregulated and downregulated, respectively), M vs. N (203 and 280 proteins were upregulated and downregulated, respectively), and RM vs. N (404 and 477 proteins were upregulated and downregulated, respectively). The results indicated that infection with MDV resulted in fewer changes in the global proteome of CEF cells than did infection with REV. Further, in RM compared with R (RM/R), 1,023 proteins that were differentially accumulated were identified, 547 of which were upregulated and 476 of which were downregulated. Of the 362 proteins identified between RM and M (RM/M), 188 and 174 proteins were upregulated and downregulated in RM, respectively (Figure 3A; Supplementary Table 1). The results showed that coinfection with MDV and REV significantly altered the abundance of proteins. To identify the common and specifically changed proteins between RM and M, between RM and R, or between M and R, a Venn diagram was generated (Figure 3B; Supplementary Table 2). It clearly showed that differentially abundant proteins (DAPs) were divided into 11 clusters obtained across all groups and divided into 11 clusters. These proteins were annotated using the GO database: molecular functions, biological processes, and cellular components (Supplementary Table 1).

The DAPs between the sample groups were defined as those with a ≥1.2-fold or ≤ 0.83-fold change in relative abundance (*p* < 0.05). An average protein ratio <1 represented downregulated proteins, and an average proteins ratio >1 represented upregulated proteins.

### Gene Ontology Enrichment Analysis

**Figure 4:**
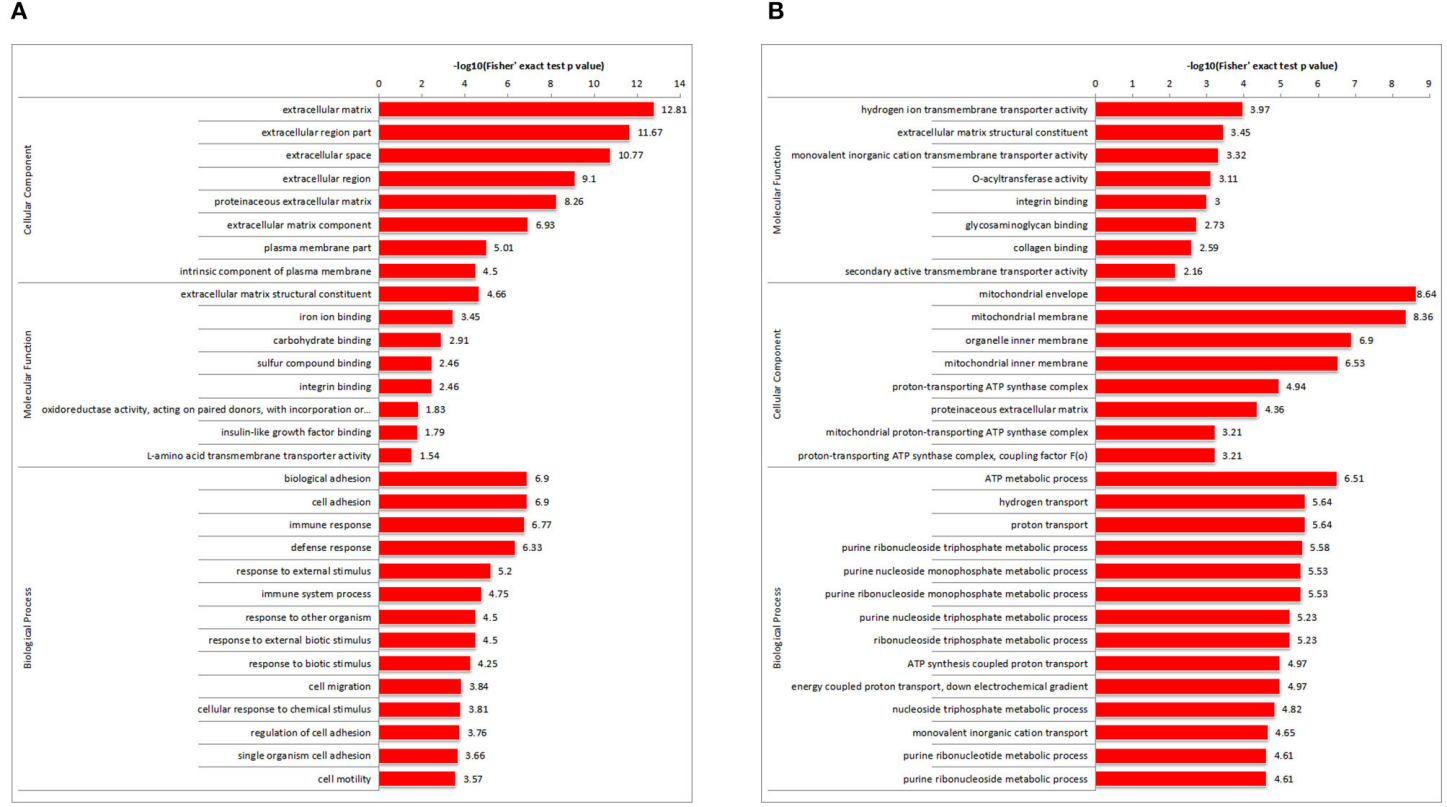
GO enriched histogram of DAPs. Statistics of GO enrichment in RM vs. M **(A)** and RM vs. R **(B)**. Each column in the figure is a GO term, the vertical axis text indicates the name and classification of GO, and the height of the column indicates the enrichment rate. R, REV-infected CEF cells; M, MDV-infected CEF cells; RM, REV and MDV coinfected CEF cells. GO, Gene Ontology; DAPs, differentially accumulated proteins; REV, reticuloendotheliosis virus; CEF, chicken embryo fibroblast; MDV, Marek's disease virus.

To determine the DAP expression trends in the different GO functional classifications, comparative cluster analysis was performed in RM vs. M and RM vs. R. For DAPs in RM vs. M (Figure 4A; Supplementary Table 3), extracellular matrix (ECM) structural constituent was highly represented in molecular function. Consistently, most DAPs were clustered in the ECM in the cellular component category. Furthermore, in terms of biological process annotation, biological adhesion process, cell adhesion, and cell migration process were enriched. Other proteins involved in immune response and immune system process were also enriched. For DAPs in RM versus R (Figure 4B; Supplementary Table 4), monovalent inorganic cation and monovalent inorganic cation transmembrane transporter activity were highly represented in molecular function. The mitochondrial membrane and organelle inner membrane were enriched in the cellular component category. ATP metabolic process, hydrogen transport, and proton transport showed significant enrichment.

### Kyoto Encyclopedia of Genes and Genomes Pathway Analysis

**Figure 5:**
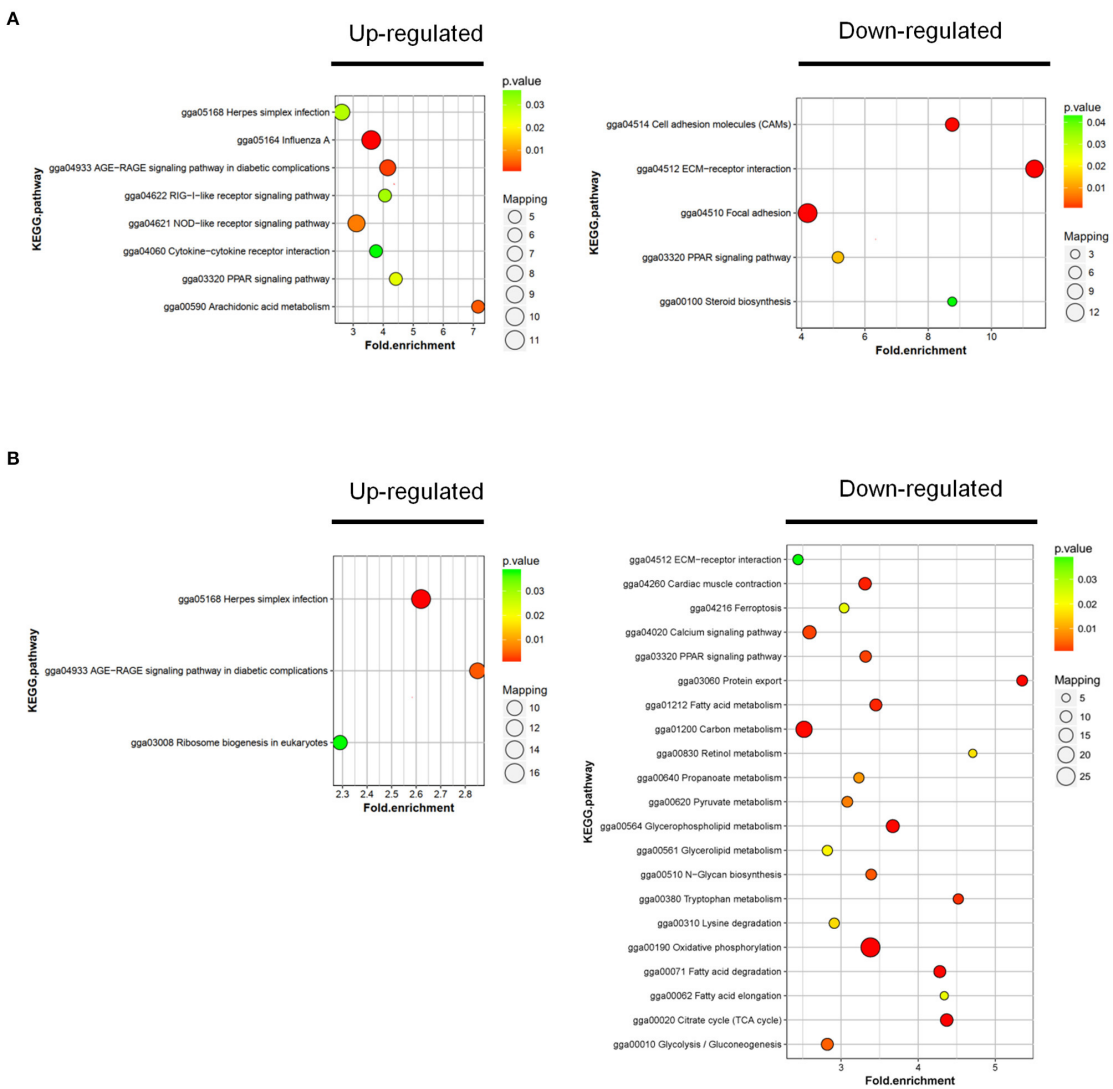
KEGG cluster and pathway enrichment analysis of DAPs. Statistics of KEGG enrichment in RM vs. M **(A)** and RM vs. R **(B)**. The pathway enrichment statistical analysis was performed by Fisher's exact test. The x-axis is folded enrichment; the y-axis is enrichment pathway. The mapping is the protein number. The color indicates the significance of the enrichment (*p*-value). The darker the color, the more significant the enrichment of the pathway. R, REV-infected CEF cells; M, MDV-infected CEF cells; RM, REV and MDV coinfected CEF cells. KEGG, Kyoto Encyclopedia of Genes and Genomes; DAPs, differentially accumulated proteins; REV, reticuloendotheliosis virus; MDV, Marek's disease virus.

To further investigate the function of DAPs between the sample groups, we analyzed the changes of rich clustering classes in KEGG pathways. In RM vs. M, the upregulated proteins identified were mapped to a total of 8 KEGG pathways, and the downregulated proteins participated in 5 pathways (Figure 5A; Supplementary Table 5). Upregulated DAPs in RM vs. R were involved in 3 pathways, and the downregulated proteins participated in 21 pathways (Figure 5B; Supplementary Table 6). The results showed that the AGE-RAGE signaling pathway in diabetic complications, ECM–receptor interaction, and PPAR signaling pathway was enriched in RM vs. M and RM vs. R, indicating the above pathway's possible involvement in MDV and REV coinfection.

### String Analysis of the Relationships Between Selected Differentially Accumulated Proteins

**Figure 6:**
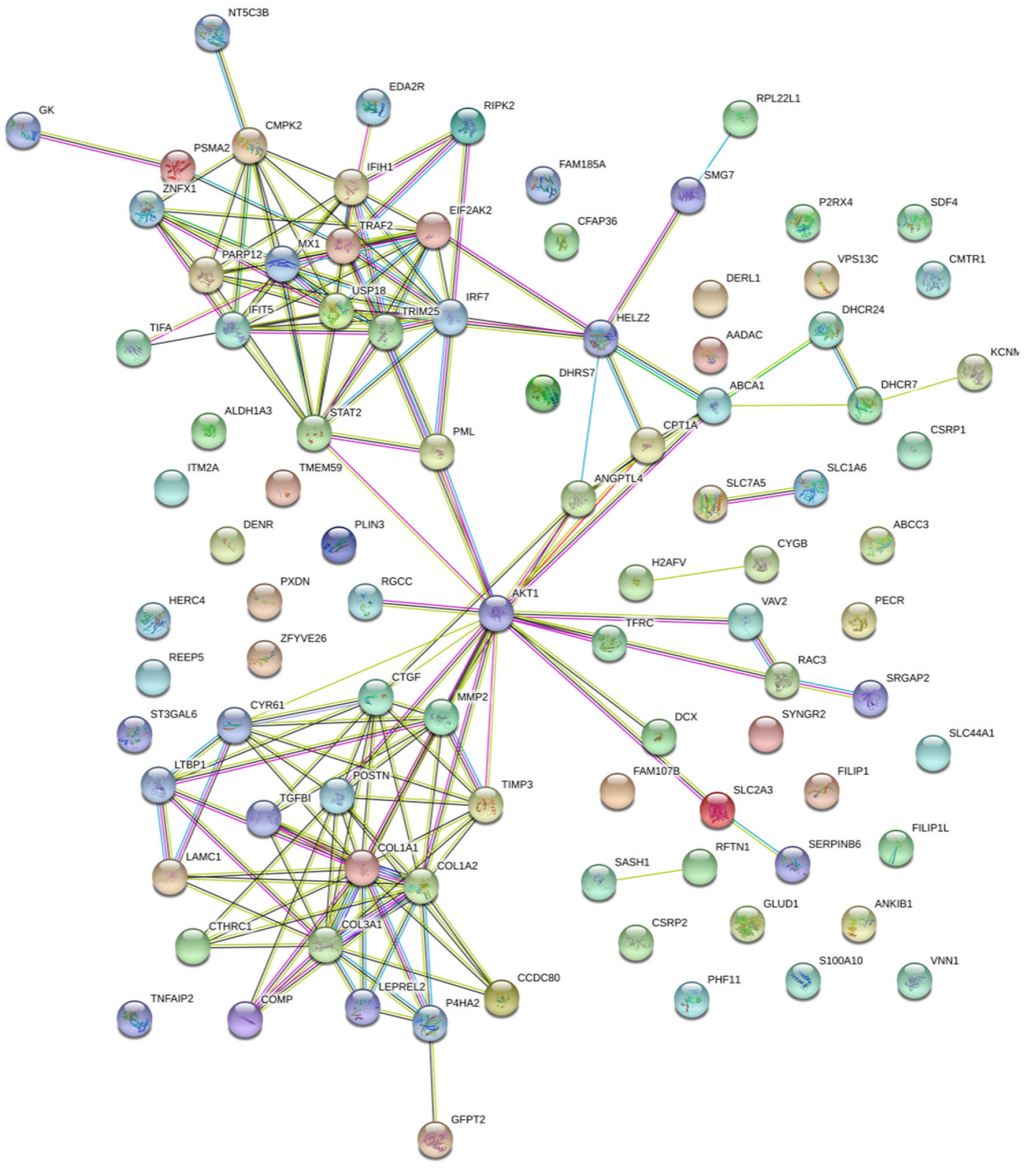
Specific network analysis of proteins significantly altered in both RM/R and RM/M. The network of DAPs with STRING analysis. Each node represents a protein in the graph, each line represents the interaction between proteins, and the wider the line, the closer the relationship. REV-infected CEF cells; M, MDV-infected CEF cells; RM, REV and MDV coinfected CEF cells. DAPs, differentially accumulated proteins; REV, reticuloendotheliosis virus; CEF, chicken embryo fibroblast; MDV, Marek's disease virus.

To further investigate, STRING tool was used to explore the potential protein network connections for the differentially regulated proteins in detail (21). In Figure 3B, of the 158 DAPs identified between RM vs. M and RM vs. R, 98 DAPs in co-expression trends were selected (Supplementary Table 7). Of these, 60 proteins were upregulated and 38 proteins were downregulated. The DAPs were mainly mapped to two functional networks by STRING software analysis (Figure 6). A specific network was focused on IRF7, IFIT5, TRAF2, MX1, TRIM25, STAT2, EIF2AK2, IFIH1, ZNFX1, CMPK2, PARP12, and USP18. These proteins were involved in innate immune pathways like the NOD-like receptor signaling pathway, RIG-I-like receptor signaling pathway, and AGE-RAGE signaling pathway. Another specific network contains AKT1, MMP2, CTGF, CTR61, LTBP1, CTHRC1, COMP, TGFβ1, LAMC1, TIMP3, POSTN, COL1A1, and COL1A2, of which ECM–receptor interaction and PPAR signaling pathway were enriched.

### Confirmation of Proteomic Data by qRT-PCR and Western Blotting Analysis

**Figure 7:**
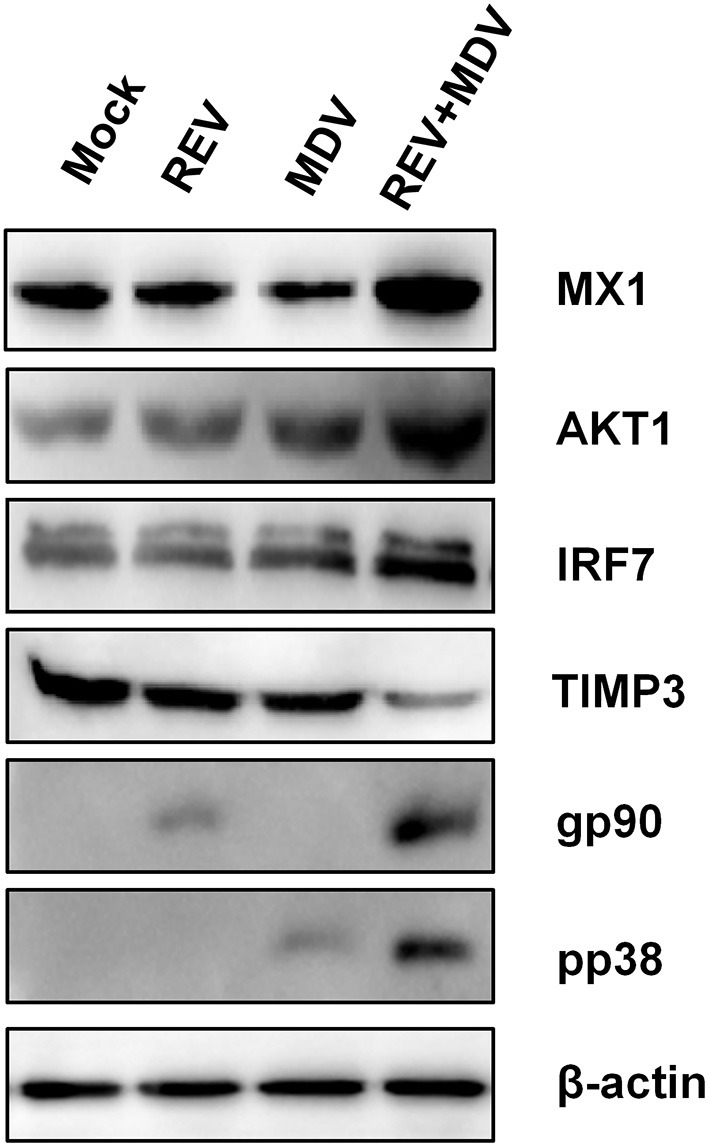
Confirmation of four differentially expressed proteins (IRF7, MX1, AKT1, and TIMP3) in MDV and REV coinfected and single infected, and mock-infected CEF cells by Western blotting. β-Actin was used as an internal control to normalize the quantitative data. MDV, Marek's disease virus; REV, reticuloendotheliosis virus; CEF, chicken embryo fibroblast.

In order to further validate the differentially expressed proteins identified by the proteomic analysis, we selected the representative proteins IRF7, MX1, AKT1, and TIMP3 for Western blotting analysis. IRF7, a crucial transcription factor to trigger innate immune responses, and Mx1, an important IFN-stimulated gene (ISG), play a pivotal role against viral infection (22, 23). It is known that activation of Akt, an important protein kinase, is crucial for the replication of many viruses (24). Furthermore, TIMPs can regulate ECM degradation and then regulate cell migration and proliferation (25). As shown in Figure 7, the expressions of IRF7, MX1, and AKT1 were obviously increased, and the expressions of TIMP3 were decreased in MDV and REV coinfected CEF cells when compared with MDV/REV-infected CEF cells. The results were consistent with those in proteomic analysis. Next, the protein expression levels of MDV and REV were quantified by Western blotting at 48 hpi. The expressions of the pp38 and gp90 proteins were significantly upregulated in the MDV and REV coinfected group.

## Discussion

Coinfection of viruses generally influences the disease pattern compared with a single infection (11, 25, 26). The outcome of coinfection usually is viral interference (27, 28). Besides interference, coinfections of viruses may also enhance viral replication and virulence (29, 30). To support virus replication, viruses can interact with a major number of cellular proteins (virus–host interactome) (31). Proteomic techniques have become significant methodologies for determining host cellular pathophysiological processes and cellular protein interactions following virus infection (32, 33). In the present study, we reported that coinfection of MDV and REV significantly increased the expression levels of both the viral gene transcript and virus titers (Figure 1). Furthermore, MDV and REV could infect in same cells and synergistically induce cytopathy (Figure 2). Taking this substantial evidence into consideration, cell samples at 48 hpi were chosen for further proteomic analysis. Based on our study, the expression levels of 98 co-regulated DAPs were found to be significantly altered upon a single infection and coinfections of MDV and REV. The results of GO, KEGG pathway, and STRING analysis predicted that these DAP pertaining to different types of functional categories and signal pathways (Supplementary Table 1). Our data may provide an overview of the proteins altered in expression during the host response to coinfection with MDV and REV may provide insight into the process of synergistic pathogenesis between two viruses.

The production of type I interferons (IFNs) is one of the most immediate responses upon infection, such as IFN-α and IFN-β (34). These secreted IFNs induce the expression of a wide range of ISGs, which collectively mediate the inhibition of viruses (35). IRF7 is an interferon regulatory factor, inducing the production of IFN-β and is crucial in the establishment of innate immunity in response to viral infection in chickens (22, 36, 37). In this study, IRF7 and MX1 were upregulated in coinfected cells (Figure 6), which indicated that MDV and REV coinfection in CEF cells leads to pronounced induction of innate immune responses in comparison to a single infection. Interestingly, some expression increased factors such as CTHRC1 (Supplementary Table 1) could be hijacked by the virus to evade host immunity and maintain replication (38, 39).

The results of this study have shown that coinfection of MDV and REV upregulated cell-adhesion molecules (CAMs) compared to a single infection (Supplementary Table 1). CAMs, a group of membrane glycoprotein and carbohydrate molecules, mediate the adhesion of cells to cells or of cells to the ECM. Virus-activated cells regulate the expression of adhesion molecules on cells in sites of infection enhanced by virus replication (40, 41). TIMP3 plays a key role in cell adhesion and migration at the cellular level and correlates with the severity of virus infection (25, 40). AKT1 upregulated upon virus infection enhances cell adhesion and decreases cell migration (42). The PI3K/Akt signal pathway is a classical phosphorylation cascade to transduce external signals to internal responses. Some viruses benefit from more than one signaling arm of the PI3K/Akt pathway (24, 43). In the present study, AKT1 was upregulated and TIMP3 downregulated at 48 hpi to various degrees following MDV and REV coinfection comparison to a single infection. This may indicate that AKT1 and TIMP3 affect the synergistic replication of MDV and REV coinfection.

The results of our proteomics assay, the large scale of proteins associated with MDV and REV single infection and coinfection, indicates that two virus synergistic replication *in vitro* interact with the innate immune pathway, Akt pathway, and cell adhesion and migration pathway, but the detailed mechanism remains unclear.

## Conclusions

Our study has provided insights into the differential manner in which the host cell proteome is regulated during single and coinfections of MDV and REV. The proteomic changes were analyzed using TMT combined with LC-MS/MS. To the best of our knowledge, this is the first time that proteomics has been used to explore the virus–host protein interaction network in MDV and REV coinfected CEF cells. The results revealed that 98 DAPs be may be associated with increased pathogenicity of MDV and REV coinfection, among which 60 were upregulated and 38 were downregulated. In addition, four DAPs were validated by Western blotting analysis. Our analyses of the DAPs were descriptive, and further functional investigations are required to elucidate the synergistic replication mechanisms and cellular responses to MDV and REV coinfection.

## Data Availability Statement

The mass spectrometry proteomics data have been deposited to the ProteomeXchange Consortium (http://proteomecentral.proteomexchange.org) *via* the iProX partner repository with the dataset identifier PXD031291. The link is https://www.iprox.cn/page/project.html?id=IPX0003983000.

## Author Contributions

ZC and XD took part in all the experiments and wrote the manuscript. ZC helped to design the whole project and draft the manuscript. DZ and JZ conducted cell culture and sample processing for sequencing and conducted data analysis. All authors read and approved the final manuscript.

## Funding

This work was supported by grants from the Shandong Modern Agricultural Technology and Industry System (No. SDAIT-11-04), the Key Research and Development Program of Shandong Province (Important Science and Technology Innovation Project) (2019JZZY010735), and the Natural Science Foundation of China (No. 32072816).

## Conflict of Interest

The authors declare that the research was conducted in the absence of any commercial or financial relationships that could be construed as a potential conflict of interest.

## Publisher's Note

All claims expressed in this article are solely those of the authors and do not necessarily represent those of their affiliated organizations, or those of the publisher, the editors and the reviewers. Any product that may be evaluated in this article, or claim that may be made by its manufacturer, is not guaranteed or endorsed by the publisher.
