# Supplementary material for: Marek's Disease Virus and Reticuloendotheliosis Virus Coinfection Enhances Viral Replication and Alters Cellular Protein Profiles
Source: Front Vet Sci. 2022 Mar 22;9:854007. doi: 10.3389/fvets.2022.854007 (PMC8981388; doi:10.3389/fvets.2022.854007)
Supplement: Supplementary file 1 [file Data_Sheet_1.ZIP › Supplementary data/Supplementary Figure S1.doc]

Figure S1. MDV and REV infection in chicken embryo fibroblasts. Detection of MDV-pp38 and REV-gp90 in infected CEFs was visualised by IFA. CEF cells infected with MDV (MOI=1) or REV (MOI=1) from 24 hpi to 72 hpi. FITC-labeled goat anti-mouse IgG (for REV) (green) and Cy3-labeled goat anti-rabbit IgG (for MDV) (red) were used as the secondary antibody in the assay.
